# Complete Genome Sequences of Two Severe Fever with Thrombocytopenia Syndrome Virus Strains Isolated from a Human and a Dog in the Republic Of Korea

**DOI:** 10.1128/MRA.01695-18

**Published:** 2019-08-01

**Authors:** Sook-Young Lee, Jun-Gu Kang, Hye-Sung Jeong, Won-Meong Kim, Ki-Dong Son, Ji Soo Kim, Sung-Suk Oh, Yoon-Kyoung Cho, Weon-Hwa Jheong, Joon-Seok Chae

**Affiliations:** a Laboratory of Veterinary Infectious Disease, College of Veterinary Medicine, Chonbuk National University, Iksan, Republic of Korea; b Laboratory of Veterinary Internal Medicine, BK21 Plus Program for Creative for Veterinary Science Research, Research Institute of Veterinary Science and College of Veterinary Medicine, Seoul National University, Seoul, Republic of Korea; c Environmental Health Research Department, National Institute of Environmental Research, Incheon, Republic of Korea; d Incheon Metropolitan City Institute of Health and Environment, Incheon, Republic of Korea; KU Leuven

## Abstract

Severe fever with thrombocytopenia syndrome virus (SFTSV) is tick-borne and causes this disease (SFTS) in humans. We determined the complete genome sequences of two SFTSV strains isolated from serum from a human with SFTS and a dog with asymptomatic infection using reverse transcription and rapid amplification of cDNA ends PCR.

## ANNOUNCEMENT

Severe fever with thrombocytopenia syndrome virus (SFTSV) belongs to the family *Phenuiviridae* and genus *Phlebovirus* and is tick-borne. The first case of SFTS was reported in China in 2011 (1), with new cases reported in South Korea and Japan in 2013 (2–4). SFTSV is an enveloped virus with a negative-sense single-stranded RNA (ssRNA) genome comprised of large (L; RNA-dependent RNA polymerase), medium (M, glycoprotein), and small (S; nucleocapsid/nonstructure protein) segments (5).

SFTSV infection is reported in not only humans and ticks but also domestic and wild animals (6–8). However, among animals, entire genome sequences of SFTSV have been reported from only hedgehog, goat, rodent, weasel, and cheetah (9–13). In this paper, we isolated and completely sequenced two SFTSV strains. The first strain was isolated from the serum of an SFTS patient, and the second was isolated from a PCR-positive serum sample from a dog (without prominent clinical signs) resulting from a surveillance study. This study was approved by the Medical Research Ethics Committee of Gacheon University Hospital (institutional review board number GBIRB2014-314).

SFTSV was isolated in monolayer-cultured Vero cells (KCLB 10081) via addition of 200-μl SFTSV-positive sera and confirmed in infected cells after 5 to 7 days via NP gene amplification according to previously described methods (14). Briefly, viral RNA was extracted from infected cells using the TRI reagent (Molecular Research Center, USA). cDNA was synthesized using the PrimeScript first-strand cDNA synthesis kit (TaKaRa, Japan) according to the manufacturer’s instructions. The three genome segments were amplified by reverse transcription-PCR using the 11 primer pairs listed in Table 1. The 5′- and 3′-end sequences of each of the two SFTSVs were confirmed via rapid amplification of cDNA ends PCR (15). Sequencing of 11 PCR products was performed using an ABI PRISM 3130 instrument (Applied Biosystem, USA). Entire genome sequences were generated from 25 sequence reads by joining overlapping sequences using ClustalW multiple alignment in BioEdit v. 7.0.9.0 (16).

**TABLE 1:** Primer sets for entire genome sequences of two SFTSV strains, KH1 and Dog22

Segment type	Primer name	Sequence	Reaction type^*a*^
S	UP-S-1	*TTTGGTCGTGGTGGTGGTTT*ACACAAAGACCCCC^*b*^	RT
	UP-S-2	*TTTGGTCGTGGTGGTGGTTT*ACACAAAGAACCCCC^*b*^	
M	UP-M-F	*TTTGGTCGTGGTGGTGGTTT*ACACAGAGACGGCCAACA^*b*^	
	UP-M-R	*TTTGGTCGTGGTGGTGGTTT*ACACAAAGACGGCCAACA^*b*^	
L	UP-L-F	*TTTGGTCGTGGTGGTGGTTT*ACACAGAGACGCCCAGAT^*b*^	
	RP-L-R	*TTTGGTCGTGGTGGTGGTTT*ACACAAAGACCGCCCAGAT^*b*^	
S	UP	*TTTGGTCGTGGTGGTGGTTT*^*c*^	PCR/Seq
	S1312R	YGTCATGAACCTGAAGGT	PCR/Seq
	S1078F	GAARACAGAGTTCACAGC	PCR/Seq
	UP-S-1/2	*TTTGGTCGTGGTGGTGGTTT*ACACAAAGA(A)CCCCC^*b*^	PCR/Seq
M	UP	*TTTGGTCGTGGTGGTGGTTT*^*c*^	PCR/Seq
	M1032R	TCYAGTGTTGCCATCATTCT	PCR/Seq
	M813F	KTGTTCWGAATCAGAAGAAA	PCR/Seq
	M2408R	CCAGCCTGRTTGCAGGGAGC	PCR/Seq
	M1473F	AGCTCCAGTGAAGCTAGTGTT	Seq
	M2282F	CARGTCTTCAAGGGTGTGAG	PCR/Seq
	UP-M-R	*TTTGGTCGTGGTGGTGGTTT*ACACAAAGACGGCCAACA^*b*^	PCR/Seq
L	UP	*TTTGGTCGTGGTGGTGGTTT*^*c*^	PCR/Seq
	L1070R	CCTGAGTCGGTCTTGATGTC	PCR/Seq
	L905F	CTRGARRTCAATAGATGTGA	PCR/Seq
	L2424R	CGTGAGAAYTCATGCTTCTT	PCR/Seq
	L1530F	CATTCAAGAGGAACCTAAGCA	Seq
	L2257F	GTGAACAGCTGGTACATTGG	PCR/Seq
	L3224R	CGSCCTTTGTCCATCCATGA	PCR/Seq
	L3059F	TGGGCYGCCATTTCCATGTT	PCR/Seq
	L4564R	CAGRTCYTCTGCCTTGCACC	PCR/Seq
	L4108R	CTGCTCCACCCAGTCTTC	Seq
	L4046F	GGGACAGGAAGAAGTATCA	PCR/Seq
	L5219R	ACATGGGTGTCCTCCATCAC	PCR/Seq
	L4935F	CATACACTGAGGAGTACAAG	PCR/Seq
	UP-L-R	*TTTGGTCGTGGTGGTGGTTT*ACACAAAGACCGCCCAGAT^*b*^	PCR/Seq

a RT, reverse transcriptase; Seq, sequencing.

b These primer pairs, including specific sequences for SFTSV, have a universal primer (UP) sequence at their 5′ end (in italics).

c TTTGGTCGTGGTGGTGGTTT (20 bp) is the UP.

The lengths of the L, M, and S segments of the two SFTSV strains were identified as 6,368, 3,378, and 1,746 nucleotides (nt), respectively. The G+C contents of each segment of the KH1 and Dog22 strains were 48.4% (L), 49.3% (M), and 49.1% (S) and 48.4% (L), 49.6% (M), and 48.3% (S), respectively. By BLASTn, each segment of the KH1 strain displayed the most similarity with the L (GenBank accession number KP663737; 99%), M (KP663738; 99%), and S (KP663739; 100%) sequences of the strain KAGBH5 from a patient reported in Korea in 2015 (17). Furthermore, each segment of the Dog22 strain displayed the highest similarity with the L segment of strain 16MS104 (MF094733; 99%), the M segment of strain 16KS29 (MF094766; 99%), and the S segment of strain 16KS29 (MF094791; 99%) from a patient reported in Korea, 2016 (unpublished data). These results suggest a potential interspecies transmission of SFTSV between humans and dogs, which implies that SFTSV is not absolutely host specific (14). Further epidemiological information would be required to investigate this hypothesis.

### Data availability.

The entire genome sequences of the two SFTSV strains have been deposited in GenBank under the accession numbers KY968712, MH464251, MH464252, MH491547, MH491548, and MH491549.
